# Tuberculous Meningitis, Vasculitis, and Pericarditis presented by deep coma

**DOI:** 10.12669/pjms.346.16350

**Published:** 2018

**Authors:** Sultan Abdulwadoud Alshoabi

**Affiliations:** 1 *Sultan Abdulwadoud Alshoabi, MD. Department of Diagnostic Radiologic Technology, College of Applied Medical Sciences, Taibah University, Almadinah Almunwwarah, Saudi Arabia. E-mail: alshoabisultan@yahoo.com*

**Keywords:** Medical imaging, Pericarditis, Tuberculous meningitis

## Abstract

A 32 years old male patient presented to the emergency room with complete loss of consciousness since three hours. This was after two weeks of night fever, sweating and considerable loss of weight with self-treatment by antipyretic drugs. In the last two days, the patient develops confusion and altered behavior. Clinical examination revealed high-grade fever and coma. CXR revealed mild cardiomegaly. Treatment started with intravenous fluids, antipyretics, and antibiotics. ***On the next day***, Echocardiography revealed mild Mitral regurgitation (MR), mild pericardial effusion with thickening of the pericardial membrane that suggested pericarditis. ESR was significantly elevated (57 mm/hour). ***After three days*** of treatment without improvement, Tuberculosis (TB) proposed and laboratory investigations implemented. Brain MRI T1 weighted images with Gadolinium injection revealed basal meningeal enhancement with multiple tiny cerebral granulomas.FLAIR-weighted images revealed multiple small high signal intensity foci in bilateral temporal lobes and the basal ganglia strongly suggesting vasculitis and ischemic lesions. CSF sample and culture was done, and anti-tuberculous drugs started with IV fluids, corticosteroids, and other supportive drugs. The results of CSF culture confirmed the diagnosis of Tuberculous meningitis. ***After two months*** of continuous anti-tuberculous treatment, the patient seemed to regain consciousness. The patient continued Rifampicin tab 700 mg, Isoniazid tab 350 mg, Ethambutol tab 400 mg, Pyridoxine tab 80 mg, Aspirin tab 100 mg and other supportive drugs for six months. The patient regained full health without any mental or motor disabilities.

## INTRODUCTION

Tuberculous meningitis (TBM) is the most common and the worst form of brain infection by Mycobacterium Tuberculosis. It causes disabilities or even death in more than half of the affected patients.^1^ Diagnosis of TBM remains a diagnostic problem. It is difficult to be diagnosed by clinical findings those are non-specific. Laboratory findings are not highly sensitive. Cerebrospinal fluid (CSF) acid-fast smear has a relatively low sensitivity, and CSF culture has an inherent delay. Newer diagnostic methods such as ELISA has limited availability, especially in developing countries.^2^

Medical imaging plays an essential role in the diagnosis of TBM. It has various imaging features. Obliteration of the basal cisterns by isodense or slightly hyperdense exudate is the most common radiological finding on CT.^3^ MRI is better than CT in the early stages of the disease. The imaging features of TBM include basal cisterns and leptomeningeal enhancement and parenchymal tuberculomas. Both MRI and CT are useful in the diagnosis of complications of TBM.^4^

This case study aimed to document a case of TBM that had an uncommon presentation and formed a diagnostic challenge. Excessive workup did for correct diagnosis and treatment. It was a tragedy for the family of a young man. TB is a significant financial burden for low-income families in developing countries.

## CASE REPORT

A 32 years old male patient presented to the emergency room with complete loss of consciousness since three hours after two weeks of night fever, sweating and considerable loss of weight with self-treatment by antipyretic drugs (Paracetamol and Ibuprofen). In the last two days, the patient develops confusion and altered behavior. Clinical examination of the patient revealed high-grade fever and coma. CXR revealed mild cardiomegaly with enlargement of the left atrium. Random blood sugar (RBS), Renal function tests (RFT) and Liver function tests (LFT) were unremarkable. Brain CT was unremarkable, no hemorrhagic stroke. Abdominal ultrasonography was normal. The patient admitted to the intensive care unit (ICU) and treatment started by intravenous fluids, antipyretics, and antibiotics.

**Fig. 1:**
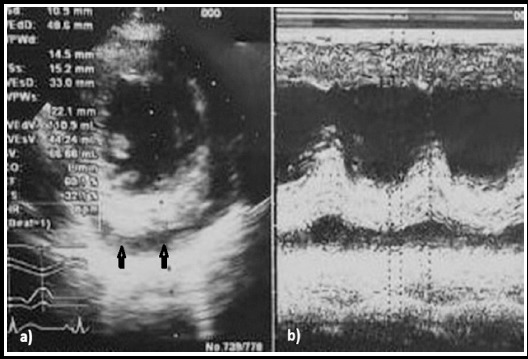
Echocardiography images a) parasternal short axis view (PSS) and b) M mode shows mild pericardial effusion (arrows) due to infective pericarditis in 32 years old patient of Tuberculous infective pericarditis.

***On the next day***, Echocardiography revealed mild Mitral regurgitation (MR), thickening of the pericardial membrane with mild pericardial effusion that suggested infective pericarditis – Fig.1. ESR was elevated significantly (57 mm/hour). The same treatment continued.

**Fig. 2:**
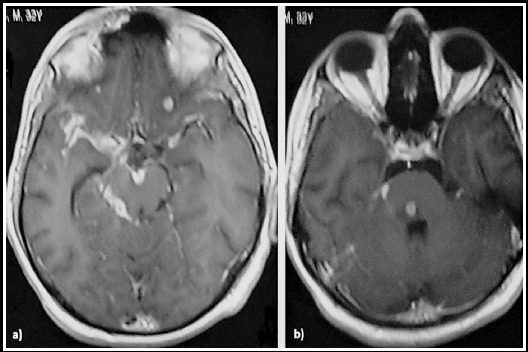
Axial Gadolinium-enhanced T1 weighted MRI images of 32 years old man show a) Meningeal enhancement that is more pronounced in the basal cisterns with small tuberculoma in the frontal lobe of the left cerebral hemisphere, b) Tuberculoma in the pons of the same patient with surrounding edema.

***After three days*** of treatment without improvement, Tuberculosis suggested and laboratory investigations implemented. Brain MRI T1 weighted images with Gadolinium revealed basal meningeal enhancement with multiple small cerebral granulomas- Fig.2.

**Fig.3:**
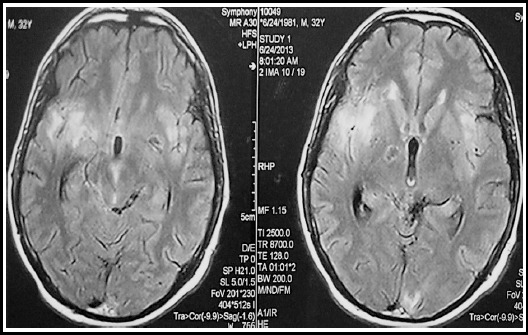
Axial FLAIR-weighted MRI images of 32 years old man shows multiple high signal intensity (SI) lesions in bilateral temporal lobes and the basal ganglia representing vasculitis and small infarctions.

FLAIR-weighted images revealed multiple tiny high signal intensity (SI) foci in bilateral temporal lobes and the basal ganglia strongly suggesting vasculitis and ischemic lesions- Fig.3.

CSF sample and culture did, and treatment started with anti-tuberculous drugs, IV fluids, corticosteroids, and other supportive drugs. The results of CSF culture confirmed the diagnosis of tuberculous meningitis.

***After two months,*** of coma with continuous anti-tuberculous treatment, the patient seemed to regain consciousness. The final diagnosis was Tuberculous meningitis, vasculitis, and pericarditis. The patient continued Rifampicin tab 700 mg, Isoniazid tab 350 mg, Ethambutol tab 400 mg, Pyridoxine tab 80 mg, Aspirin tab 100 mg and other supportive drugs for six months. The patient regained full health without any mental or motor disabilities. He didn’t remember anything about the period of coma.

## DISCUSSION

Tuberculous meningitis is one of the diagnostic challengings because of the difficulties in rapid identifying the Mycobacterium Tuberculosis in the CSF samples.^2^,^5^ The patient in the current case had a history of fever, confusion, significant weight loss before the coma. These clinical findings are consistent with Tai MLS et al. (2016), who reported that fever; confusion and weight loss were the clinical features in 76%, 65% and 33% of the patients in his study.^6^

Medical imaging plays an essential role in the early diagnosis of TBM that is a crucial point to prevent worse complications of the disease. In the current case, determination of TB was solely made based on medical imaging and the clinical history of the patient. MRI revealed enhancement of the basal cisterns and multiple small tuberculomas more evident in the frontal lobe of the left cerebral hemisphere and the pons. These findings are the typical presentation of TBM as reported by Gambhir S et al. (2017).^7^

In this cases, two tuberculomas were evident in the pons and the left frontal lobe. These findings are similar to Garg RK et al. (2016) and Sadashiva N et al. (2017), who reported that most of the cerebral tuberculomas are present in the frontal lobe and the pons respectively.^4^,^8^

They typically present as small lesion compatible with a previous study by Sadashiva N et al. (2017), who reported that most of cerebral tuberculomas size ranging from 2 to 22.2 cm^3^.^8^ However, cerebral tuberculomas can present as solitary large lesion with gyral enhancement similar to tumors as reported by Gameraddin M et al. (2016).^9^

In the current case, multiple tiny ischemic areas occur; these findings are compatible with Burrill J et al. (2007), who reported that ischemic infarctions are the common complication in TBM.^10^

In this case, Tuberculous pericarditis was present as a thickening of the pericardium with pericardial effusion. These findings are consistent with Burrill J et al. (2007), who reported that tuberculous pericarditis occurs as pericardial thickening more than 3 mm and/or pericardial effusion in some cases.^10^

The current case study includes TBM and multiple tuberculomas. These are the most common forms of brain tuberculosis consistent with two previous studies by Burrill J et al. (2007) and Ahluwalia VA et al. (2013), who reported that TBM is the most common form of brain TB and tuberculomas are the most common form of parenchymal lesions.^10^,^11^

## CONCLUSION

Tuberculous meningitis is a diagnostic problem either clinically and laboratory. Medical imaging plays an essential role in the early diagnosis of Tuberculous meningitis and pericarditis. MRI with Gadolinium is the best imaging modality for early diagnosis.
